# Psoriatic Arthritis Linked to Pembrolizumab in a Lung Cancer Patient: From Oncologist to Rheumatologist

**DOI:** 10.7759/cureus.92171

**Published:** 2025-09-12

**Authors:** Mihaela-Maria Pasca Fenesan, Elena Sirbu, Andreea Iana

**Affiliations:** 1 Microscopic Morphology/Histology, Doctoral School in Medicine, Department of Clinical Oncology, Victor Babes University of Medicine and Pharmacy, Timisoara, ROU; 2 Physical Therapy and Special Motricity, West University of Timisoara, Timisoara, ROU; 3 Family Medicine, Centre for Preventive Medicine, Victor Babes University of Medicine and Pharmacy, Timisoara, ROU

**Keywords:** disease-modifying antirheumatic drugs, immune-related adverse events (iraes), lung cancer, pembrolizumab side effect, psoriatic arthritis (psa)

## Abstract

Immune-related rheumatic adverse effects (rheumatic IAEs) are commonly observed in patients undergoing immunotherapy for solid tumors and hematologic malignancies. The Romanian National Health Insurance House offers all patients free access to oncology medications and has significantly improved in recent years.

Here, we present a case of a 69-year-old male patient diagnosed with a bronchopulmonary neoplasm who developed psoriatic arthritis (PsA) after receiving immunotherapy with pembrolizumab, emphasizing the challenges of therapeutic management in this case.

## Introduction

Lung cancer is the most prevalent cancer among men and a leading cause of death in Romania, affecting 75 per 100,000 individuals, which is 8% higher than the EU average. Among women, the lung cancer mortality rate stands at 20 deaths per 100,000, which is 33% lower than the EU average [1].

Ongoing efforts aim to improve the survival of patients diagnosed with this disease. The objective is early diagnosis and multimodal treatment based on the decisions of the multidisciplinary commission, starting from the initial stages of the disease. Lung cancer treatment relies on several factors, including the type and stage of cancer and the patient's overall health and preferences [1].

Surgery is the preferred first-line treatment for localized disease in stages I and II [2]. Stage III usually benefits from multimodal treatment, as surgical resection often results in recurrence [3]. 

Radiotherapy is the preferred treatment for patients who cannot undergo surgery, at a dose of 60 Gy over 30 fractions. For N1 tumors, chemoradiation is favored, as it offers a survival advantage compared to radiotherapy alone. Stereotactic radiotherapy can be a viable option in T1-2N0M0 cases but is not recommended for tumors that are located less than 2 cm from the proximal bronchial tree or for those greater than 5 cm in diameter. Stereotactic radiotherapy achieves a similar disease control rate as surgery for tumors with a diameter of less than 3 cm [2,3].

Adjuvant treatment for non-small cell lung cancer (NSCLC) aims to eliminate residual cancer cells after surgery, thereby lowering the risk of recurrence. Several factors, including cancer stage, molecular markers, and patient health, influence adjuvant therapy selection. Cytotoxic chemotherapy, particularly cisplatin-based regimens, has remained the standard adjuvant treatment for early-stage NSCLC. It has shown survival benefits, especially in stage II and III patients [2,3].

Recent advances have introduced immunotherapy as a viable adjuvant option to enhance the immune system's response to cancer [4].

Currently, the most commonly used immunotherapy regimens include pembrolizumab (Keytruda), durvalumab (Imfinzi), nivolumab (Opdivo), and atezolizumab (Tecentriq) [4,5].

Pembrolizumab (Keytruda) is a monoclonal antibody that the US Food and Drug Administration (FDA) approved in January 2023 for adjuvant treatment following surgery and platinum-based chemotherapy in patients with stage IB (T2a ≥4 cm), II, or IIIA NSCLC [6-8].

Immunotherapy, or immune checkpoint blockade therapy (ICBT), exerts its antitumor effect by blocking cytotoxic T lymphocyte antigen 4 (CTLA-4) and programmed cell death 1 (PD-1). PD-1 is a checkpoint protein found on T and B cells, which is essential for regulating the immune system. It is highly expressed on tumor-specific T cells and binds to two other proteins, the PD-L1 and PD-L2 ligands. When PD-1 binds to PD-L1, it prevents the activation of T cells, which are responsible for destroying other cells, including cancer cells [9]. Although the aforementioned anticancer drugs that block the PD-1 axes induce an antitumor response, they are associated with immune-related adverse effects (IAEs). The pathophysiology of these adverse effects is not fully understood, as most published studies have been case studies or small case series.

The most frequently reported immune-related adverse effects include rashes, colitis, hepatitis, pancreatitis, iridocyclitis, inflammatory arthritis, lymphadenopathy, neuropathies, and nephritis [10,11].

This paper discusses the challenges of therapeutic management for a patient with bronchopulmonary neoplasm and pembrolizumab-induced psoriatic arthritis (PsA).

## Case presentation

A 69-year-old male smoker was admitted to Department of Pneumology, Victor Babes Hospital in Timisoara in March 2020, with an intermittent dry cough, fatigue, and erythematous-squamous plaques on the anterior surface of the right calf. 

The patient had a 50-pack-year smoking history and was exposed to respiratory toxins due to his profession (sewing industry). The patient's sister and son had been diagnosed with psoriasis vulgaris. His medical history included hypertension, chronic obstructive pulmonary disease GOLD (Global Initiative for Chronic Obstructive Lung Disease) stage 2, chronic coronary syndrome, and psoriasis. He was taking antihypertensives (indapamide 1 tablet per day, nebivolol 5 mg daily), aspirin 75 mg daily, and Seretide 500 mcg/50 mcg.

The respiratory symptoms appeared two to three weeks prior to his admission, and because his condition worsened, he had imaging studies in March 2020. 

**Figure 1:**
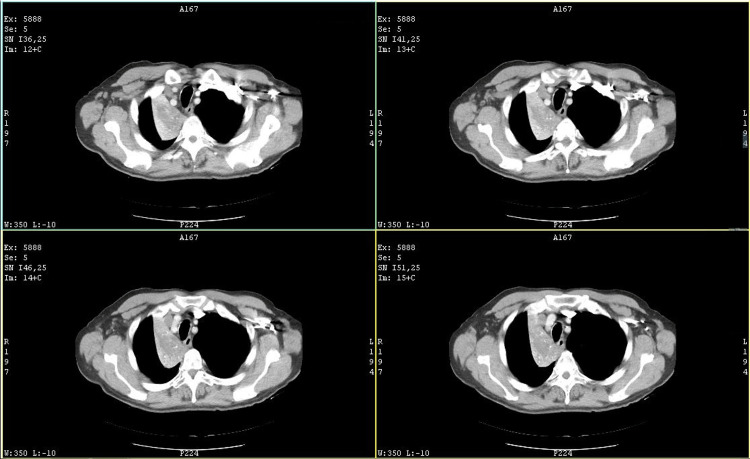
Initial CT scan thorax

**Figure 2:**
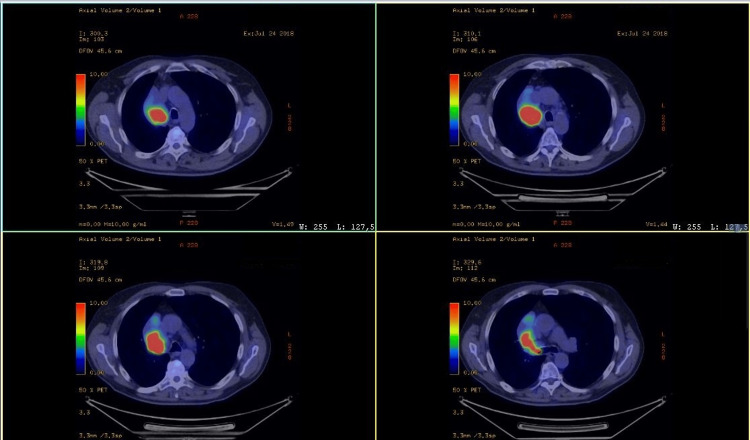
Initial PET CT scan thorax PET: Positron emission tomography

A CT scan revealed complete atelectasis of the right lower lung lobe and a 56/39mm formation suggestive of a proliferative process, along with several lymph nodes in the right paratracheal space and in the aorto-pulmonary window, measuring up to 8 mm in the short axis (Figures 1, 2).

Further investigations were requested, leading to a fiberoptic bronchoscopy. The bronchoscopy showed a vegetative proliferative process extending from the apical segment of the lobe and prolapsing into the lumen. Histopathological examination and immunohistochemistry demonstrated a non-keratinized squamous carcinoma (TTF1 negative, p63 positive).

The confirmed diagnosis of bronchopulmonary neoplasm stage IIB, with specific characteristics cT3N0M0, was established, and treatment was initiated using carboplatin AUC6 along with paclitaxel 175 mg/m^2^ every three weeks to reduce the tumor and prepare for surgery. Platinum-based chemotherapy is the standard treatment for NSCLC. At the time of our patient's diagnosis, there was no other standard neoadjuvant treatment approved in our country. 

Following four cycles of chemotherapy, a right upper lobectomy with bronchoanastomosis was performed in August 2020.

The patient's histopathological examination indicateCs squamous cell lung carcinoma (G2 grade, Pn1, LV1, pT3N1M0). Genetic testing reveals negative results for Epidermal Growth factor receptor mutation (EGFR) and anaplastic lymphoma kinase rearrangement (ALK), with a PD-L1 tumor proportion score (TPS) of 5%.

Two months later, a new CT scan showed that the bilateral pulmonary nodular lesions had increased in both number and size compared to the previous CT examination. 

**Figure 3:**
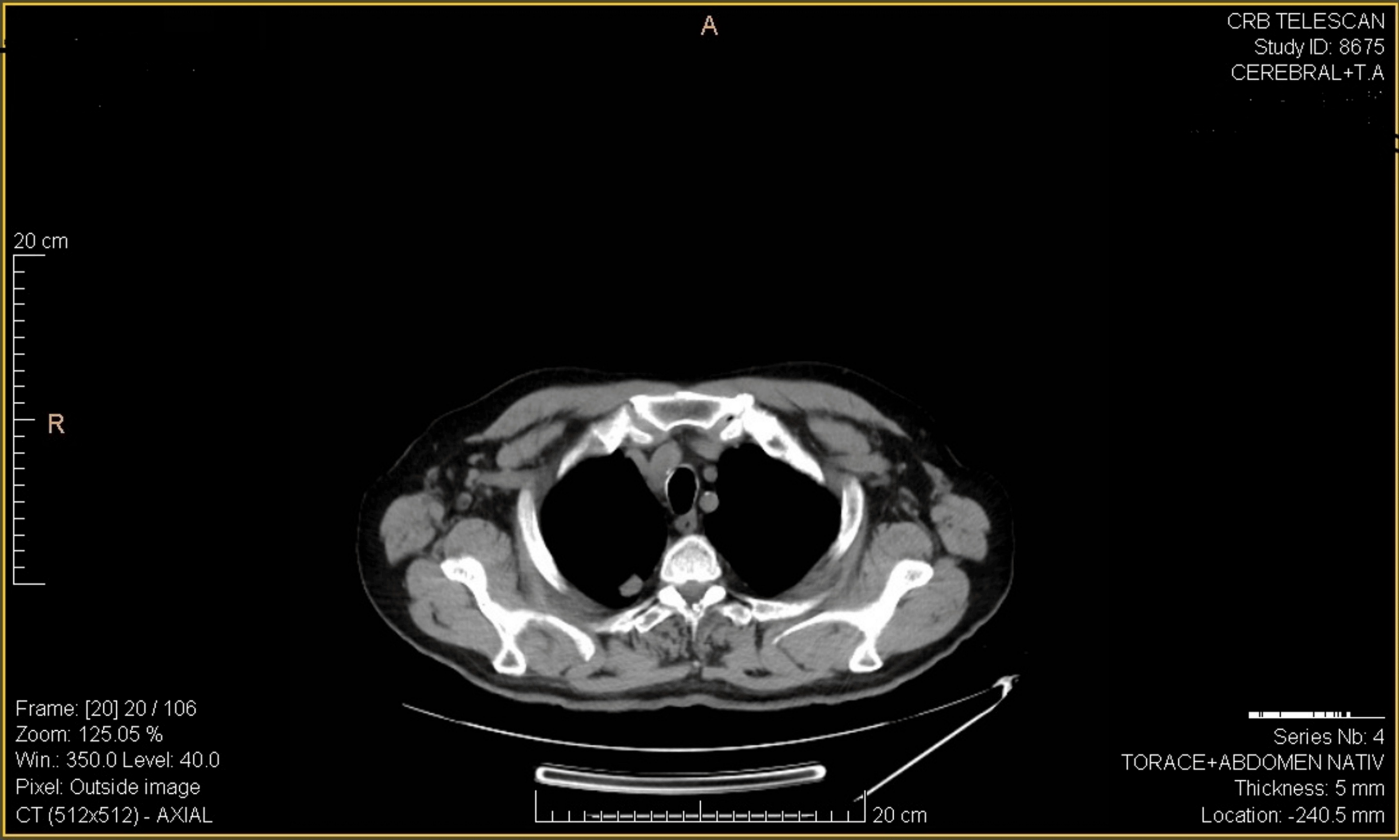
CT scan showing subpleural nodular lesion posterior to the right inferior lobe

**Figure 4:**
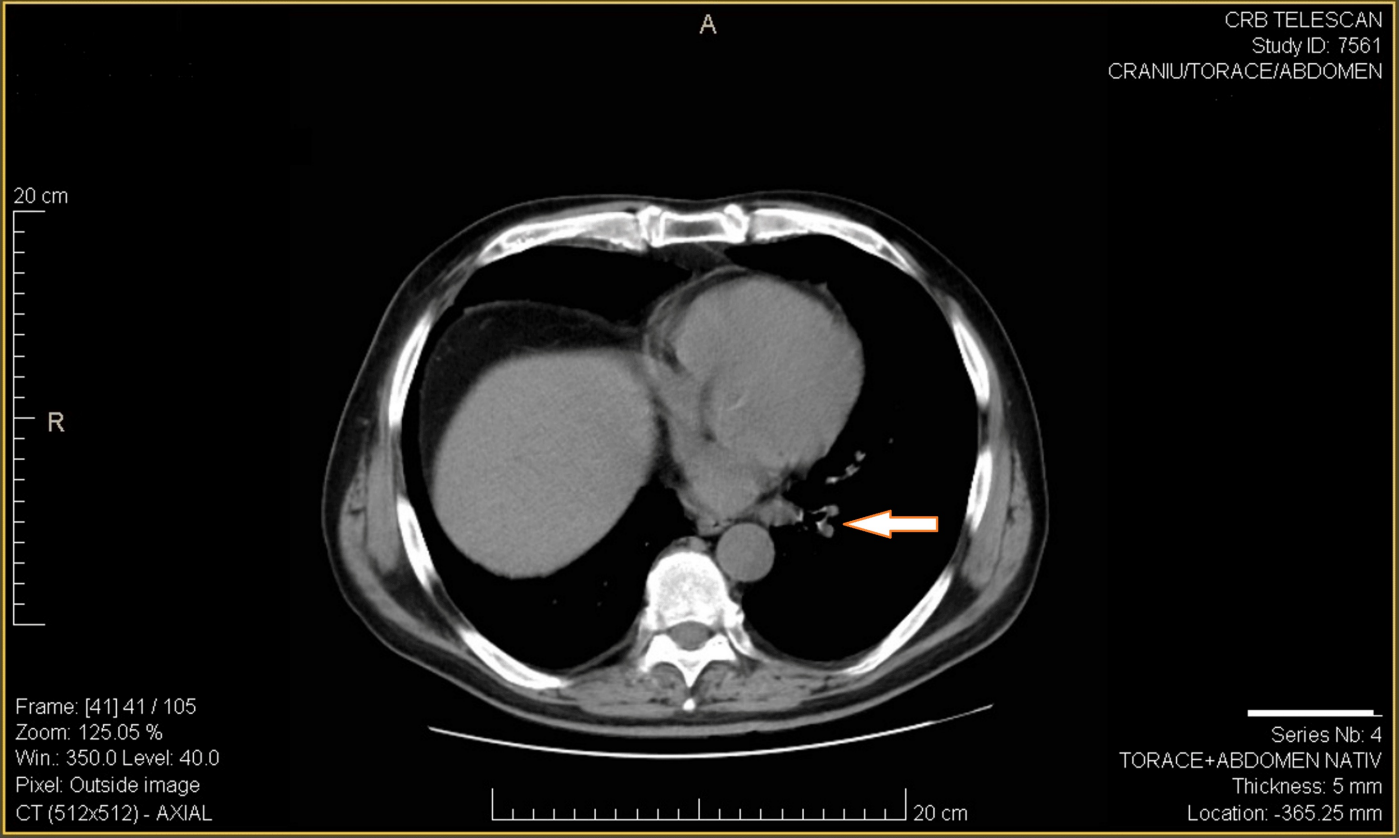
CT scan showing the "de novo" appearance of two left nodular lesions

Moreover, this CT examination, revealed a new subpleural nodular lesion posterior to the right inferior lobe (LIR), exhibiting moderately increased contrast uptake (Figure 3). It also showed the "de novo" appearance of two left nodular lesions (Figure 4). 

At that moment, pulmonary progression is assessed, resulting in the decision to resume chemotherapy (four cycles of paclitaxel and carboplatin) and to combine therapy with pembrolizumab. We considered re-administering paclitaxel and carboplatin based on the negative PD-L1 expression shown by the genetic tests.

After, completing 12 cycles of pembrolizumab, we observed a reduction in the size of the nodular lung lesions (1.2/0.9 cm vs 1.7/1.3 cm), although the patient developed eyelid edema. 

The endocrinology consultation confirmed the diagnosis of thyroiditis, and treatment with Euthyrox (112.5 mcg/day) was started.

Cycles 13-24 of pembrolizumab were well tolerated for 10 months until a repeat CT of the head, thorax, and abdomen indicated an increase of over 30% in the subpleural nodular postero-basal lesion compared to the previous CT scan. Multiple psoriatic skin lesions were identified, and the dermatologist prescribed a non-steroidal topical treatment containing anthralin.

**Figure 5:**
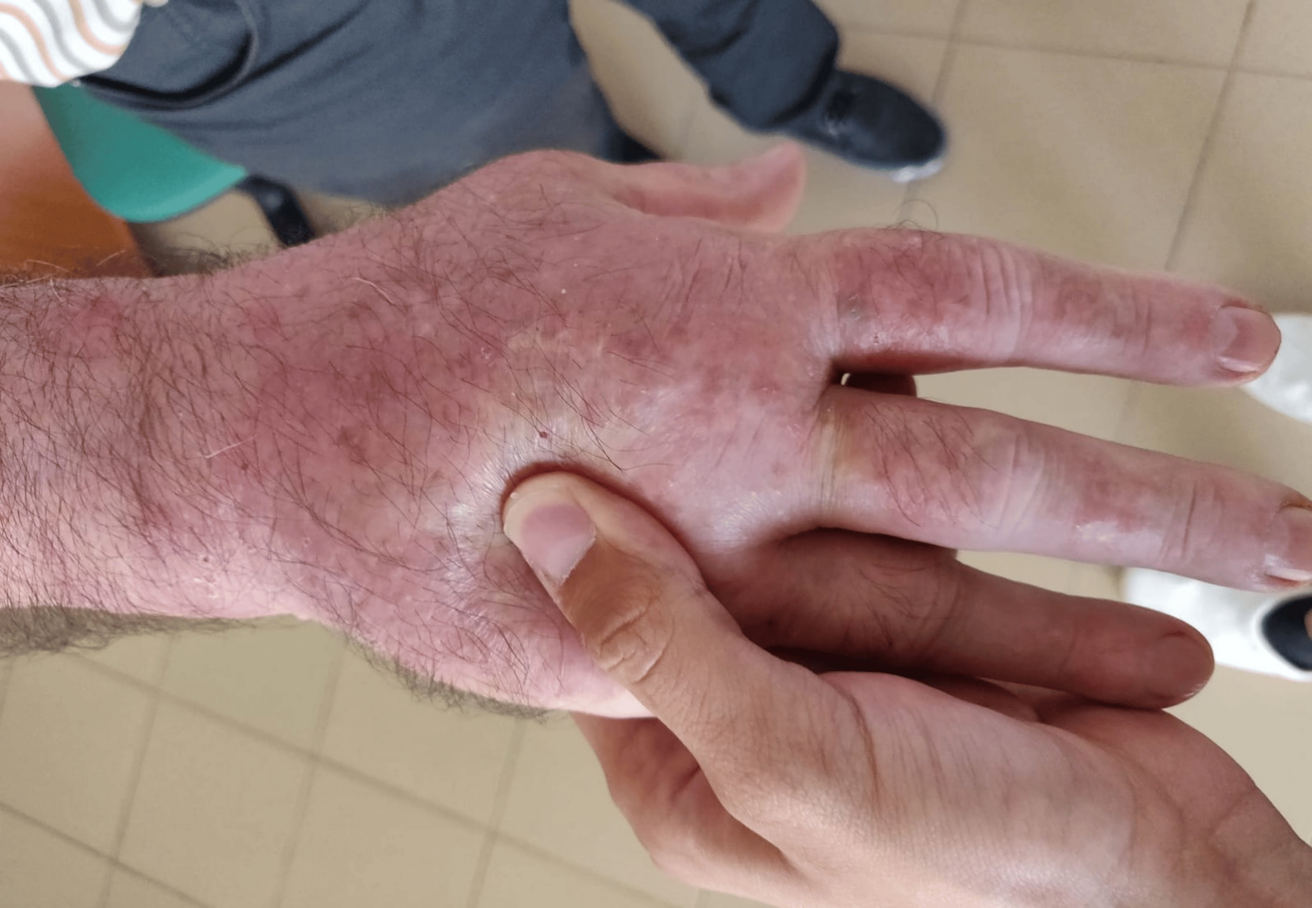
Psoriatic polyarthritis showing hand swelling

**Figure 6:**
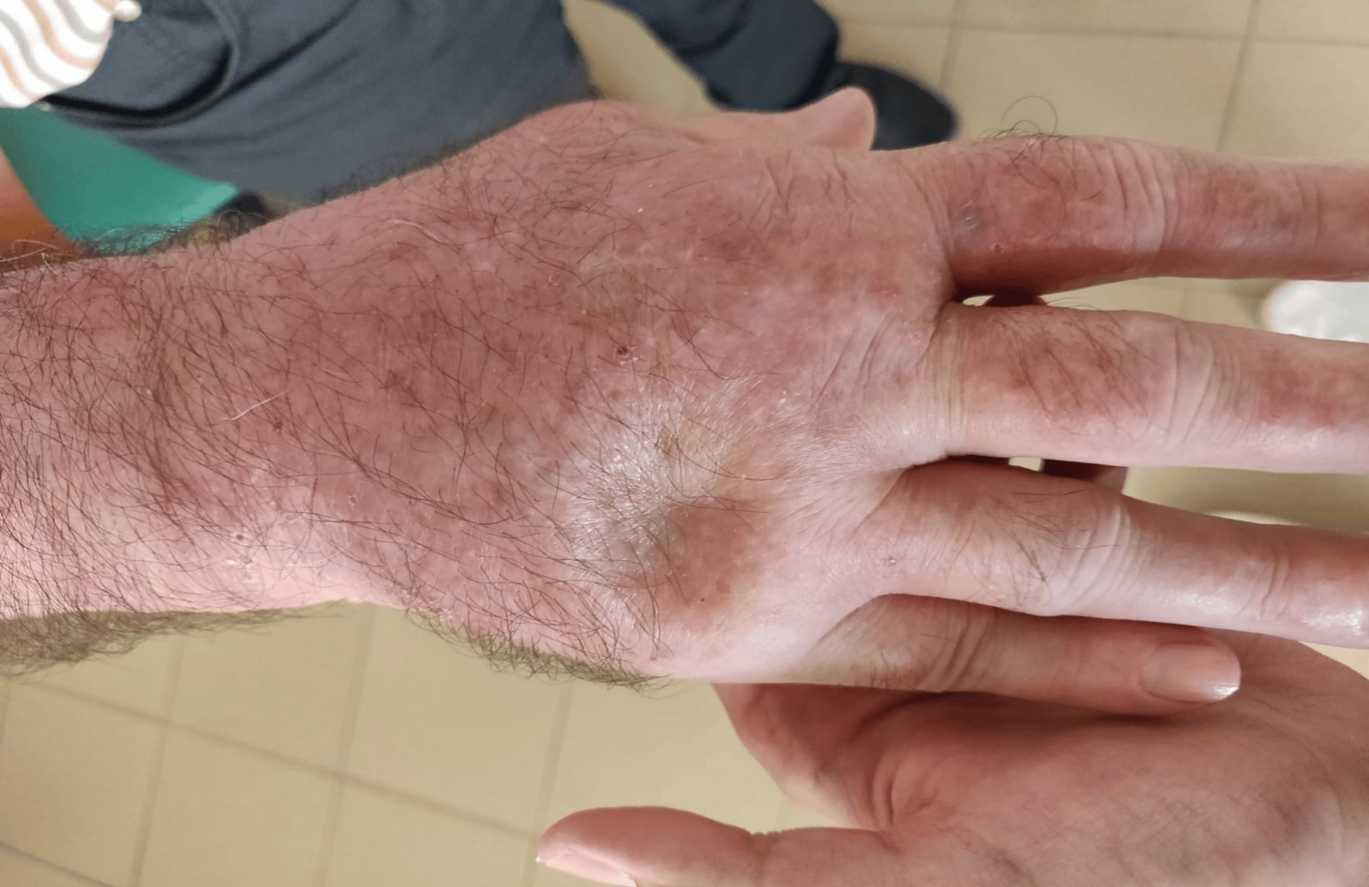
Pitting test for hand edema

After 24 cycles of pembrolizumab, he started to experience joint pain in his hands, shoulders, and ankles. He presented to the rheumatology office with joint pain and swelling in his hands and ankles, accompanied by stiffness (Figures 5, 6).

**Figure 7:**
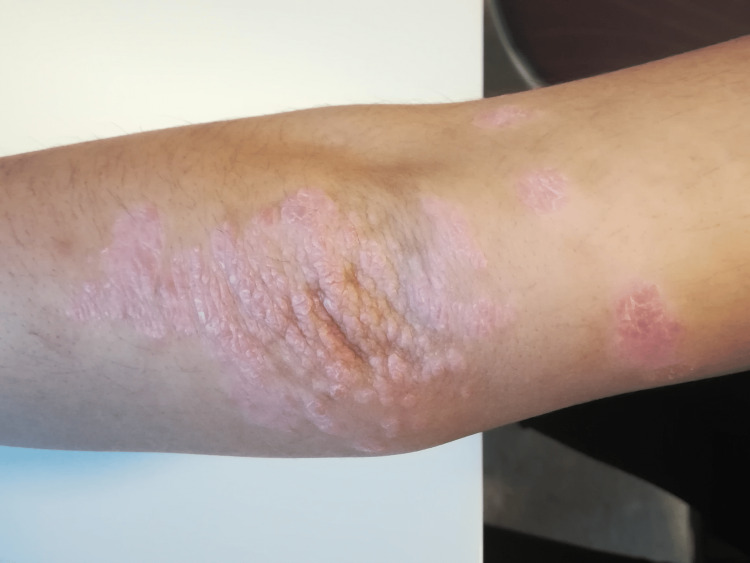
Psoriasiform rash on the right elbow

Upon admission, his physical examination revealed a psoriasiform rash on the elbows (Figure 7), on his right leg, abdomen, and anterior aspect, associated with musculoskeletal complaints, limited shoulder mobility, and crepitus during knee mobilization. He reported difficulty performing daily activities due to restricted joint mobility in his hands and shoulders. Further laboratory tests and imaging studies were advised.

Laboratory tests indicated anemia, with hemoglobin levels at 10.2 g/dl, elevated C-reactive protein at 34.7 mg/L, erythrocyte sedimentation rate at 63 mm/h, negative rheumatoid factor at 9.9 UI/ml, anticitrullinated cyclic peptide at 0.5 UI/ml, antinuclear antibodies absent, and human leukocyte antigen B27+ (Table 1).

**Table 1:** Laboratory findings

Variable	Value	Reference range
White blood cell count, cells/mm^3^	11.77/mm^3^	4.23-9.07/mm^3^
Neutrophiles, %	74.4%	34-67.9%
Monocites, %	8.1%	5.3-12.2%
Red blood cell count, cells/mm^3^	4.80/mm^3^	4.63-6.08/mm^3^
Hemoglobin, g/dl	10.2 g/dl	13.7-17.5 g/dl
Platelet count, cells/mm^3^	432,000/mm^3^	150,000-450,000/mm^3^
Erythrocyte sedimentation rate, mm/h	63 mm/h	0-20 mm/h
C-reactive protein, mg/L	34.7 mg/L	0-5 mg/L
Glucose, mg/dl	112 mg/dl	<125 mg/dl
Creatinine, mg/dl	0.87 mg/dl	0.67-1.17 mg/dl
Rheumatoid factor, UI/ml	9.9 UI/ml	<14 UI/ml
Antinuclear antibodies	Negative	Negative
Anticitrullinated cyclic peptide, UI/ml	0.5 UI/ml	<5 UI/ml
Human leukocyte antigen B27	Positive	Negative

**Figure 8:**
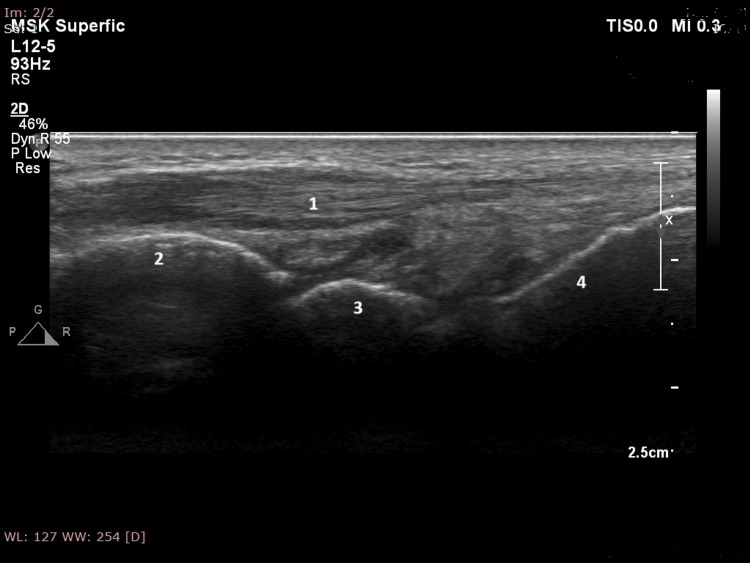
Extensor tenosynovitis in the third finger of the right hand 1: Extensor tendon of the third finger; 2: Capitate; 3: Lunate; 4: Radius

The musculoskeletal ultrasound showed arthritis in the radiocarpal joints on both sides and extensor tenosynovitis in the third finger of the right hand (Figure 8).

According to the Classification for Psoriatic Arthritis (CASPAR) criteria, the patient was diagnosed with moderate PsA (Disease Activity in Psoriatic Arthritis (DAPSA) score: 16) and started on sulfasalazine (SSZ) at a dose of 4x500 mg/day, progressively increasing, along with omeprazole 20mg/day. He noticed a partial improvement in symptoms despite persistent inflammatory biologic syndrome. Corticotherapy was initiated using oral prednisolone at a daily dose of 15 mg for two weeks, tapered progressively to 5 mg/day, and SSZ at 6x500mg/day.

After this treatment, the patient showed improvement with the disappearance of arthritis and edema (DAPSA score: 10). However, a new CT scan revealed an increase in the left basal pulmonary nodular lesion size after Cycle 33 of pembrolizumab.

In this situation, curative hypofractionated radiation therapy using the volumetric modulated arc therapy (VMAT) technique and 6 MV photons for the pulmonary nodule, delivering a total dose of 45 Gy in 15 fractions (3 Gy per fraction), was recommended. The remaining cycles of pembrolizumab (34-41) were carried out without incident. The patient was discharged from our service with SSZ 6x500mg/day.

Additional medication included esomeprazole 40 mg orally, taken daily, along with daily supplementation of calcium and Vitamin D. The patient’s outcome over the next two years was unfavorable, marked by the progression of cerebral and pulmonary lesions in June 2022. During this time, he underwent palliative external radiotherapy to the skull, receiving a dosage of 30 Gy over 10 fractions, and paclitaxel chemotherapy. Unfortunately, the patient passed away in January 2023 due to cerebral disease progression.

## Discussion

Studies are examining the effectiveness of combining chemotherapy with immunotherapy. For instance, postoperative adjuvant chemotherapy alongside immunotherapy has demonstrated significant effectiveness compared to chemotherapy alone, improving disease-free survival and overall survival rates. Most experts suggest a multimodal approach should be considered: immunotherapy combined with chemotherapy, both perioperatively (neoadjuvant therapy) and postoperatively (adjuvant treatment). Studies such as the AEGEAN (a phase III, double-blind, placebo-controlled, multi-center international study of neoadjuvant/adjuvant durvalumab for the treatment of patients with resectable stages II and III non-small cell lung cancer), KEYNOTE-671 (neoadjuvant pembrolizumab plus chemotherapy followed by adjuvant pembrolizumab compared with neoadjuvant chemotherapy alone in patients with early-stage non-small cell lung cancer), and NEOTORCH (perioperative toripalimab plus chemotherapy for patients with resectable non-small cell lung cancer) have shown that a “sandwich” model of neoadjuvant-surgical-adjuvant therapy significantly enhances patient survival [12-15].

Unlike those previously mentioned, our 69-year-old male patient diagnosed with stage IIB lung cancer underwent the following treatment regimen: perioperative chemotherapy, surgery, and combined immunotherapy (pembrolizumab), along with postoperative chemotherapy.

The decision to use adjuvant therapy should be personalized, considering the potential benefits and risks. Consulting with an oncologist is essential for identifying the most appropriate treatment plan based on the latest evidence and individual patient factors.

The outcomes were favorable after receiving 12 cycles of pembrolizumab, showing a reduction in the size of the nodular lung lesions; however, he developed thyroiditis secondary to immunotherapy. Additionally, after 24 cycles of pembrolizumab, our patient reported joint pain in his hands, shoulders, and ankles. He was assessed as having PsA as an IAE and received SSZ 4x500 mg/day.

In PsA, joint symptoms can occur before, after, or alongside skin symptoms. Most patients initially develop psoriasis, but a significant minority experience arthritis before or at the same time as skin symptoms. Joint symptoms often appear years after psoriasis begins. Our patient developed joint symptoms after 24 cycles of pembrolizumab, within one year and four months.

Certainly, considering the patient's family history, it is difficult to determine whether the arthritis is caused by the natural progression of psoriasis or as a side effect of immunotherapy. After reviewing the literature and the patient's progress, we considered it to still be a side effect of immunotherapy.

To reduce disease activity, the dose of SSZ was subsequently increased to 6×500 mg per day, and a corticosteroid therapy of 15 mg daily was added. After two weeks, the corticotherapy was progressively tapered to 5 mg daily.

In this decision, we considered that corticosteroid therapy is viewed as a prohibited medication during treatment, and it is essential to pause immunotherapy if high doses of cortisone are required [16].

Similar to our study, most cases reported in the literature indicate that oncologic patients treated with various immune checkpoint inhibitors experienced rheumatic manifestations classified as IAEs [17,18].

In a study by Lidar et al., rheumatic manifestations were identified in 14 of 400 patients (3.5%) who underwent immunotherapy. The most common rheumatic manifestation was inflammatory arthritis (85%), with one-third of patients having predisposing factors, including a personal or family history of psoriasis, a previous episode of uveitis, or positive anti-CCP antibodies [19]. Another study published by Gonzalez-Mazón et al. reported 87 patients with immune-related adverse events, primarily gastrointestinal, endocrine, and musculoskeletal manifestations, such as arthralgia, arthritis, and myositis [20].

It is important to note that our case presented with a family history of psoriasis and minimal lesions on the elbows. 

Given the moderate joint involvement and the severity of the oncological disease, it was decided to continue treatment with pembrolizumab, weighing the benefits as significantly greater than the potential risks.

Additionally, because the joint disease was controlled, it was unnecessary to combine another traditional disease-modifying antirheumatic drug (DMARD) or to initiate biological DMARD therapies. However, in severe or refractory cases, it is advisable to consider either synthetic or biological DMARDs.

In this regard, it is essential to note that early referral to a rheumatologist facilitates better disease control and prevents the reactivation of PsA. Close interdisciplinary collaboration enabled the remission of joint symptoms, the administration of the most appropriate rheumatic medication, and the resumption of oncological treatment when the acute episode of PsA was in remission. Pembrolizumab was continued with careful rheumatological follow-up, allowing the patient to benefit from the latest standard of oncological treatment.

Our study has a limitation that should be acknowledged. Although the case presentation provides sufficient clinical and imaging details, the image from the histopathological examination trough biopsy would strengthen the case description. However, due to circumstances beyond our control, we could not obtain the image from the histopathological examination, which was conducted at another university center, but we received the interpretation from the pathologist.

## Conclusions

IAEs in cancer are continuously increasing, necessitating a multidisciplinary team of medical oncologists, rheumatologists, and immunologists. Nonsteroidal anti-inflammatory drugs (NSAIDs), corticosteroids, and DMARDs are the preferred therapeutic options for managing inflammatory arthritis. However, their effects on the efficacy of antitumor therapies and the best drug combinations require thorough evaluation.
